# Polyphenols and IUGR Pregnancies: Intrauterine Growth Restriction and Hydroxytyrosol Affect the Development and Neurotransmitter Profile of the Hippocampus in a Pig Model

**DOI:** 10.3390/antiox10101505

**Published:** 2021-09-22

**Authors:** Natalia Yeste, Néstor Gómez, Marta Vázquez-Gómez, Consolación García-Contreras, Martí Pumarola, Antonio González-Bulnes, Anna Bassols

**Affiliations:** 1 Departament de Bioquímica i Biologia Molecular, Facultat de Veterinària, Universitat Autònoma de Barcelona, Cerdanyola del Vallès, 08193 Barcelona, Spain; natalia.yeste@uab.cat (N.Y.); nestor.gomez@uab.cat (N.G.); 2 Faculty of Veterinary Sciences, UCM, Ciudad Universitaria s/n, 28040 Madrid, Spain; mvgomez@ucm.es (M.V.-G.); antonio.gonzalezbulnes@uchceu.es (A.G.-B.); 3 Comparative Physiology Group, INIA, Avda. Puerta de Hierro s/n, 28040 Madrid, Spain; garcia.consolacion@inia.es; 4 Unitat de Patologia Murina i Comparada, Departament de Medicina i Cirurgia Animals, Facultat de Veterinària, Universitat Autònoma de Barcelona, Cerdanyola del Vallès, 08193 Barcelona, Spain; marti.pumarola@uab.cat; 5 Departamento de Producción y Sanidad Animal, Facultad de Veterinaria, Universidad Cardenal Herrera-CEU, CEU Universities, Tirant lo Blanc, 7, Alfara del Patriarca, 46115 Valencia, Spain

**Keywords:** hydroxytyrosol, neurotransmitters, intrauterine growth restriction, hippocampus, brain, pig

## Abstract

Intrauterine growth restriction (IUGR) refers to poor growth of a fetus during pregnancy due to deficient maternal nutrition or oxygen supply. Supplementation of a mother’s diet with antioxidants, such as hydroxytyrosol (HTX), has been proposed to ameliorate the adverse phenotypes of IUGR. In the present study, sows were treated daily with or without 1.5 mg of HTX per kilogram of feed from day 35 of pregnancy (at 30% of the total gestational period), and fetuses were sampled at day 100 of gestation. Fetuses were classified as normal body weight (NBW) or low body weight (LBW) as a consequence of IUGR, constituting four groups: NBW-Control, NBW-HTX, LBW-Control, and LBW-HTX. The brain was removed, and the hippocampus, amygdala, and prefrontal cortex were rapidly dissected. Neuronal markers were studied by immunohistochemistry, and a decrease in the number of mature neurons in the hippocampal *Cornu Ammonis* subfield 1 (CA1) and the *Dentate Gyrus* (DG) regions was observed in LBW fetuses together with a higher number of immature neurons and other alterations in neuronal morphology. Furthermore, IUGR conditions altered the neurotransmitter (NT) profile, since an increase in the serotonin (5-HT) pathway was observed in LBW fetuses. Supplementation with HTX was able to reverse the morphological and neurochemical changes, leading both characteristics to values similar to those of NBW fetuses.

## 1. Introduction

The effect of intrauterine growth restriction (IUGR, also known as fetal growth restriction, FGR) is characterized by a deficit in growth and weight of the individual due to a lack of nutrients or oxygen or an alteration in the placenta that can lead to a lack of these essential components for development [1]. In IUGR, the “brain-sparing” effect occurs, in which blood flow to the brain increases to ensure its development. That is, there is an asymmetric growth of the individual in such a way that brain development is prioritized to ensure the maintenance of the critical physiological functions of the newborn, such as breathing, suckling, and other autonomic functions, which improve the chances of vitality and survival. This effect has been described in different species of mammals, including pigs, sheep, humans, and guinea pigs [1,2].

However, “brain-sparing” does not completely guarantee the normal development of the brain or its function, since it has been described that the offspring affected by IUGR (low body weight, LBW) can suffer from behavioral disorders related to mobility and cognitive, memory, and neurophysiological dysfunctions [3,4]. The animal models studied have shown that individuals affected by IUGR presented alterations in the hippocampus with consequences in normal neurological development [5]. IUGR is also associated with excessive production of reactive oxygen species (ROS), which may occur at specific windows of placental development and, for these reasons, supplementation of maternal diets with antioxidants (e.g., antioxidant vitamins and melatonin) has been investigated as a potential way to ameliorate adverse phenotypes [6,7,8,9,10]. Hydroxytyrosol (HTX) is a potent antioxidant, present in olive fruits and virgin olive oil, that presents regulatory properties in metabolism, inflammation, and immunomodulation and also acts as neuroprotector in human pathologies and animal models [11,12,13,14]. The beneficial effects of HTX are mainly attributed to its potent antioxidant and ROS scavenger activities, which are able to counteract the pernicious consequences of oxidative stress in the organism. Furthermore, other potential mechanisms with a direct effect on regulatory enzymes such as p38 and JNK have been proposed [11].

Caloric restriction in sows during the third trimester of gestation affects fetal development and induces lower birth weight in newborns, being a well-known animal model of IUGR widely used by our groups in previous studies [15,16]. In this model, the NBW and LBW animals are born from the same mothers, thus avoiding the effect of the mother in the statistical evaluation of the results.

In previous studies from our laboratories, maternal HTX supplementation in this porcine model was associated with higher mean birth weight and lower incidence of low-birth-weight piglets. The positive effects of HTX administration were sustained during lactation, leading to increased body weight at weaning [17]. It also resulted in deviations in body composition and metabolic indices, suggesting increased potential for growth and viability that was confirmed in a later study [18]. Later, the effects of HTX on fetal antioxidant status, placental gene expression, and fat metabolism were demonstrated [19,20,21]. Nonetheless, maternal supplementation with HTX during pregnancy also influenced brain parameters since it affected the neurotransmitter (NT) profile in a brain-area-dependent mode and it modified the process of neuron differentiation in the hippocampal *Cornu Ammonis* subfield 1 (CA1) and *Dentate Gyrus* (DG) areas, indicating that cell differentiation occurred more rapidly in the HTX group than in the control group [22]. These effects were specific to the fetal period, concomitantly with HTX maternal supplementation, since no major differences were detected in 1-month- and 6-month-old pigs.

Since the effects of maternal HTX supplementation on neurotransmission and hippocampal morphology were observed only in 100-day fetuses [22], we decided to evaluate whether the effects of HTX were influenced by the body weight of the offspring. Thus, the specific goals of the present work were to study whether the body weight of the fetuses (NBW or LBW) could differentially influence the morphological analysis of the hippocampus and the NT levels in various areas of the brain, and if HTX supplementation of the maternal diet could partially or totally reverse the effects of IUGR.

## 2. Materials and Methods

### 2.1. Ethics Statement

The study was carried out at the INIA animal facilities, which meet local, national, and European requirements for Scientific Procedure Establishments, and was performed according to the Spanish Policy for Animal Protection RD53/2013, which complies with the European Union Directive 2010/63/UE on the care of animals used for research. The experimental procedure was assessed and approved by the INIA Committee of Ethics in Animal Research (report CEEA 2013/036).

### 2.2. Animals and Experimental Procedure

The study involved 13 purebred Iberian sows that became pregnant after cycle synchronization with altrenogest (Regumate, MSD, Boxmeer, The Netherlands) and insemination with cooled semen from a purebred Iberian boar.

The sows were fed with a standard grain-based food diet adjusted to fulfil individual daily maintenance requirements based on data from the British Society of Animal Science [23]. On gestational day 35, all sows were weighed and the food amount from that day until delivery was adjusted to fulfil 50% of daily maintenance requirements. This diet restriction has previously been found to affect fetal development and to induce a higher incidence of low birth weight in newborns [24,25]. Additionally, on gestational day 35, sows were pair-matched according to bodyweight and seven females remained as an untreated control group (CTRL), whilst the six remaining females acted as the treated group by receiving 1.5 mg of HTX per kg of feed each day from day 35 of pregnancy to delivery (HTX).

Fetuses were obtained at day 100 of pregnancy (55 Ctrl and 44 HTX). Immediately after retrieval, all fetuses were sexed and weighed and classified into two groups: low body weight (LBW) and normal body weight (NBW). LBW fetuses were defined by a weight lower than one standard deviation of the mean value of the littermates after adjusting for sex. This weight threshold is commonly used to identify IUGR effects and offspring at a higher risk of perinatal mortality [17]. Applying these criteria, 10 and 6 LBW fetuses were classified in the CTRL and the HTX group, respectively.

Sampling was performed after stunning and exsanguination in compliance with RD53/2013 standard procedures. Subsequently, the head was separated from the trunk at the atlanto-occipital union and the brain was removed from the skull and weighed. Both hippocampi, both amygdalae, and the prefrontal cortex were dissected. One of the hippocampi, one of the amygdalae, and the prefrontal cortex were snap-frozen in liquid nitrogen and biobanked at −80 °C until neurotransmitter quantification. The remaining hippocampus was fixed in 4% buffered formalin in PBS (Amresco, Solon, OH, USA) for 24 h at 4 °C, preserved in 30% sucrose in PBS at 4 °C, and used for immunohistochemistry.

### 2.3. Sample Preparation and Neurotransmitter Quantification

Samples were weighted and homogenized by sonication (Branson Digital Sonifier 250, Branson Ultrasonics Corp., Danbury, CT, USA) in a lysis buffer (150 mM NaCl, 50 mM Tris-HCl, and 1% NP-40) with a 0.3 mg tissue/mL of lysis buffer relation. Dihydroxybenzylamine (DHBA) was added to the lysis buffer at 100 pg/µL as an internal standard for HPLC. Proteins in brain lysates were precipitated by adding 0.25 M perchloric acid containing 0.1 M sodium metabisulfite and 0.25 M EDTA in a 1.5 (*v*/*v*) ratio. Finally, samples were centrifuged at 12,000× *g* for 10 min at 4 °C and kept at −80 °C until analysis.

Concentrations of catecholamines (NA, DA, DOPAC, and HVA) and indoleamines (5-HT, 5-HIAA) were determined by HPLC (EliteLaChrom, Merck-Hitachi, Prague, Czech Republic) equipped with a Cromolith Rp-18e column (Merck, Darmstadt, Germany) with electrochemical detection (ESA Coulochem II 5200, Thermo Scientific, Braunschweig, Germany). The mobile phase consisted of 0.05 M citrate buffer pH 2.8, 0.05 mM EDTA, 1.2 mM sodium octyl sulphate (SOS), and 1% acetonitrile. The applied voltage was set at 0.4 mV and the flow rate was 1.2 mL/min. All procedures are described in detail in [26].

### 2.4. Immunohistochemical Analysis of the Hippocampus

LBW individuals (10 in the CTRL group and 6 in the HTY group) were matched with NBW individuals. Litter, sex, and weight were considered when choosing the NBW fetuses. Therefore, from the same mother, another individual of the same sex with the highest weight was chosen (Supplementary Table S1). Using these selection criteria, 20 animals from the CTRL group were obtained: 10 LBW (5 females and 5 males) and 10 NBW (5 females and 5 males); and 12 animals from the HTX group: 6 LBW (3 females and 3 males) and 6 NBW (3 females and 3 males).

Hippocampal samples were frozen in OCT medium (Aname, Madrid, Spain) using molds, an isopentane bath (Sigma, St. Louis, MO, USA), and dry ice, controlling the freezing temperature between −40 °C and −60 °C. The OCT blocks were cut with a cryostat (SME Cryotome Thermo Electron Corporation, Thermo Scientific, Braunschweig, Germany) into 40-μm-thick sections in a longitudinal orientation, collecting them in flotation with an antifreeze solution pH 7.4 (40% ethylene glycol, 30% glycerol, and 30% phosphate buffer 0.1 M pH 7.4).

For immunohistochemistry, a minimum of 6 sections per individual were analyzed. Sections were washed using a phosphate buffer 0.1 M pH 7.4, and endogenous peroxidase activity was blocked using 1% H_2_O_2_. Sections were blocked with 2% normal goat serum (NGS) and incubated with the corresponding primary antibodies with NGS overnight at 4 °C. The antibodies used were raised against NeuN (1:1000, Mouse monoclonal anti-neuronal nuclei; Merck Millipore, Chemicon, Billerica, MA, USA, Ref. MAB377), doublecortin (DCX, 1:750, Rabbit polyclonal anti-doublecortin; Abcam, Cambridge, MA, USA, Ref. ab18723), and neurofilaments (NFT, 1:10,000, Mouse monoclonal anti-neurofilament 200; Sigma, St. Louis, MO, USA, Ref. N0142). Afterward, the sections were washed and incubated with biotinylated goat secondary antibodies (1:500, anti-mouse IgG or anti-rabbit IgG; Agilent Technologies, Dako, Glostrup, Denmark). Next, sections were incubated with an avidin–biotin–peroxidase complex (Standard ABC Peroxidase Staining Kit; Pierce Biotechnology, Rockford, IL, USA) and revealed with 3,3′-diaminobenzidine tetrahydrochloride (DAB Liquid Substrate System; Sigma, St. Louis, MO, USA). Sections were transferred to Superfrost Plus™ adherent slides, counterstained with hematoxylin, and mounted in resinous DPX mounting medium (Sigma, St. Louis, MO, USA).

### 2.5. Image Processing and Analysis

Slides were digitally scanned with a 2.0 HT Nanozoomer (Hamamatsu Photonics, Hamamatsu, Japan) at the Histopathology Service of the Biomedicine Research Institute (IRB, Barcelona, Spain). The scanned images were visualized and analyzed using NDP.view 2 software (Hamamatsu Photonics, Hamamatsu, Japan). Only CA1 and DG were analyzed since those were the only areas affected by HTX treatment in our previous work [22].

NeuN immunostaining was analyzed using ImageJ 1.52 p software (NIH, Bethesda, MD, USA) from the website of the National Institutes of Health. The procedure performed to analyze the images was: convert the scanned color image to grayscale (8-bit); set the measurement scale; threshold the image using “Make Binary”; and finally analyze particles. The entire tissue area, neuron area, and neuron area percentage were calculated. To obtain the level of the specific DAB signal on all of the tissue in the photograph, the actual area of neurons was calculated by subtracting the blank areas that contained no tissue (e.g., lumina of vessels and artifacts). Moreover, the DAB-positive area outside the neuron area was excluded. Individual neuron clusters were numbered to obtain information regarding a particular neuron (size, circularity, area, etc.) from the tabulated results.

### 2.6. Statistical Analyses

All statistical analyses were performed in SPSS 24.0 software (IBM, Chicago, IL, USA). The significance level was established at *p* < 0.05 and a tendency was considered at 0.05 ≤ *p* ≤ 0.1. Descriptive data are presented with the means and the standard error (mean ± SE).

Normal distribution of the variables was confirmed with a Kolmogorov–Smirnov test. Whenever possible, data were log transformed to correct the distribution and hence permit use of parametric statistics.

Normally distributed measures were analyzed using the UNIANOVA procedure of SPSS with Tukey’s adjustment. In all models, each pig was introduced as the experimental unit, and the fixed effects included were treatment (CTRL and HTX), body weight (NBW and LBW), sex (male and female), and their interactions. In addition, pairwise comparisons with Bonferroni’s adjustment were also performed for significant interactions.

## 3. Results

### 3.1. Effects of Maternal Supplementation with HTX on Immunohistochemical Markers in the Hippocampus of NBW and LBW Fetuses 

Antibodies against NeuN, DCX, and NFT, markers of mature neurons, immature neurons, and neurofilaments, respectively, were used to analyze the effects of IUGR and HTX on the morphology and development of the hippocampus.

**Figure 1:**
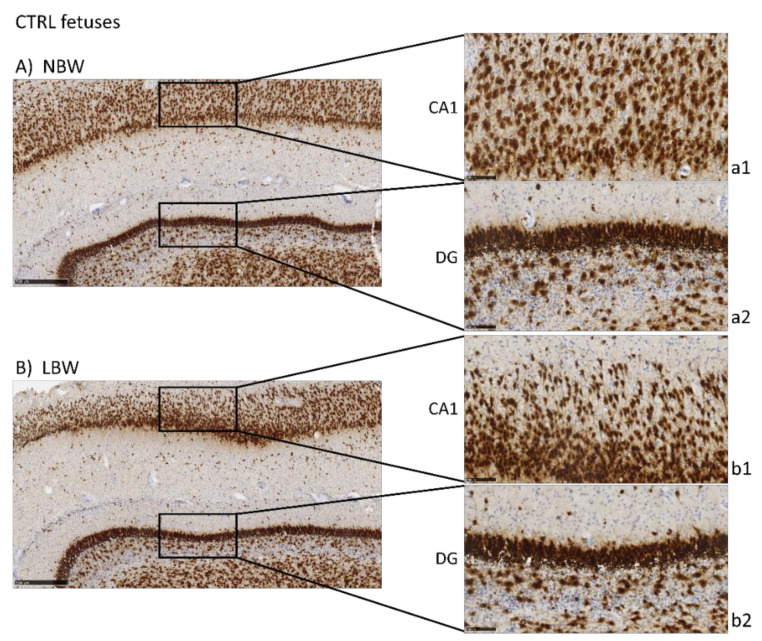

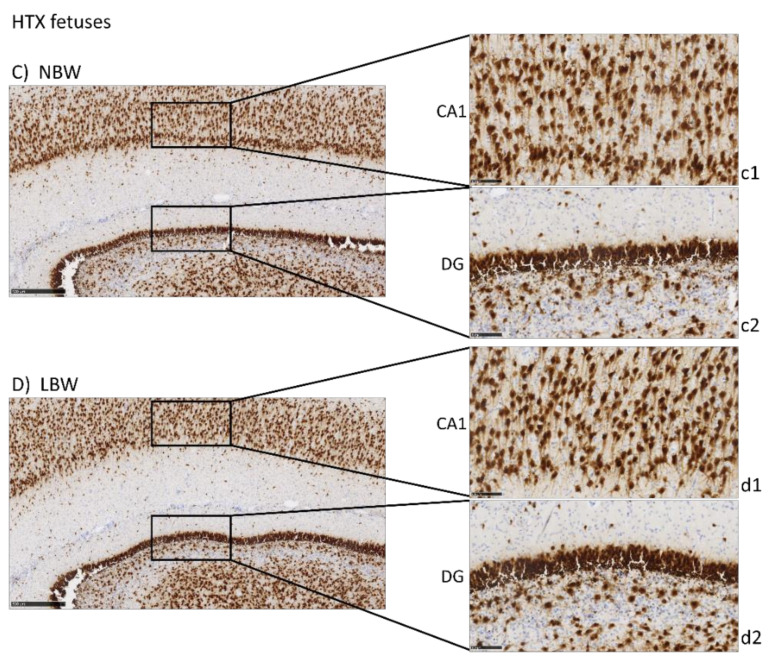
Effect of Intrauterine growth restriction (IUGR) on NeuN immunostaining on the hippocampus of fetuses whose mothers were not HTX-supplemented (**A**,**B**) or were HTX-supplemented (**C**,**D**). Representative images show the mature neurons immunostained with the NeuN antibody. Panels are magnifications of the CA1 (**a1**–**d1**) and GD (**a2**–**d2**) areas shown using black boxes. Scale bars: 500 µm (**A**–**D**), and 100 µm (**a1**–**d1**, **a2**–**d2**).

NeuN is a marker of neuronal bodies and nuclei in mature neurons and, thus, NeuN staining was used to analyze the number and distribution of mature neurons in CA1 and DG areas in the hippocampus of 100-day fetuses and to assess the effects of IUGR and HTX (Figure 1). Higher positivity was observed in the NBW fetuses than in the LBW fetuses, thus indicating that the NBW animals had a higher number of mature neurons. The quantitative results obtained after image analysis are presented in Table 1. Quantitatively, the effect of weight was significant only in CA1, but not in DG, indicating that IUGR affected mainly this region. The cell count was lower in LBW fetuses, with a higher mean cell nuclei size and area percentage.

In the pairwise comparison, it is interesting to note that the differences between NBW and LBW in CA1 were only observed in fetuses whose mothers were supplemented with the control (cell count, *p* < 0.001; mean size, *p* < 0.001; and area, *p* = 0.001), while they were not observed when the mothers were supplemented with HTX (cell count, *p* = 0.580; mean size, *p* = 0.971; and area, *p* = 0.679).

Regarding the effects of maternal supplementation, HTX treatment increased the cell number and decreased the mean nuclei size in CA1, especially in LBW fetuses (cell count, *p* < 0.001; mean size, *p* < 0.001; and area, *p* < 0.001), while this effect was much less significant in the NBW fetuses (cell count, *p* = 0.027; mean size, *p* = 0.139; and area, *p* = 0.069).

In DG, there were no differences due to body weight and the effects of HTX were the same as in CA1, i.e., there was an increase in the cell number and a decrease in the mean nuclei size in both NBW and LBW fetuses. In the pairwise comparison, it can be observed that the effects on LBW fetuses were in general more significant than in NBW animals (LBW fetuses: cell count, *p* = 0.152; mean size, *p* < 0.001; and area, *p* < 0.001 and NBW fetuses: cell count, *p* = 0.135; mean size, *p* = 0.030; and area, *p* = 0.025). Thus, the neuronal characteristics of the HTX-treated LBW group became more like those of the NBW animals, especially the HTX-treated NBW group, and the differences between NBW and LBW observed in control animals disappeared or were less relevant in HTX-treated animals.

**Figure 2:**
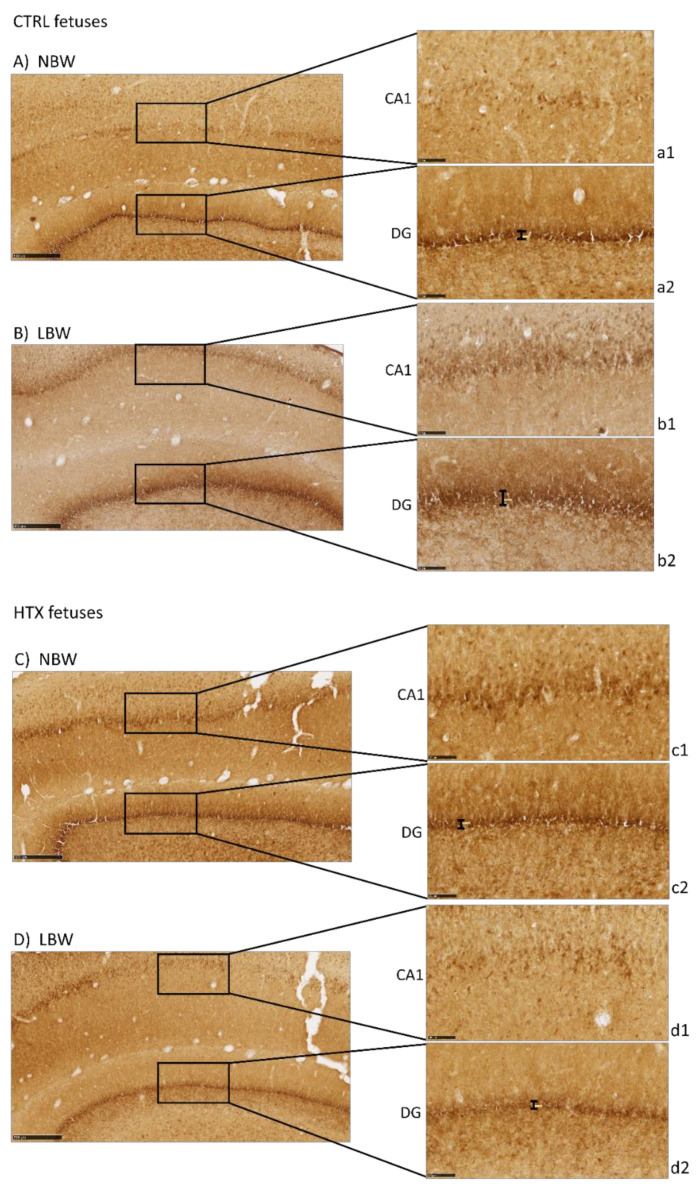
Effect of IUGR on DCX immunostaining on the hippocampus of fetuses whose mothers were not HTX-supplemented (**A**,**B**) or were HTX-supplemented (**C**,**D**). Representative images show the immature neurons immunostained with the DCX antibody. Panels are magnifications of the CA1 (**a1**–**d1**) and DG (**a2**–**d2**) areas shown using black boxes. Scale bars: 500 µm (**A**–**D**), and 100 µm (**a1**–**d1**, **a2**–**d2**). The thickness of the DG area is indicated by a black bar.

Precursor neuronal cells (neuroblasts) were visualized during their migration and differentiation with doublecortin protein staining (DCX), which is associated with microtubules. As can be observed in Figure 2, LBW fetuses presented more intense staining in the CA1 area than the NBW fetuses, thus indicating that there was a greater number of immature neurons. It has to be noted that the excessive staining background did not allow for the quantification of DCX-positive neurons. These results were inverse to the results obtained with NeuN staining, indicating that these regions in the hippocampus of LBW individuals contained fewer mature neurons concomitantly with a higher number of immature cells. Similar results were observed in DG. Treatment of the mothers with HTX produced LBW individuals who presented weaker DCX immunostaining in CA1 as well as in DG areas, leading to a situation more similar to that of NBW control individuals.

In addition, the thickness of the DG area was measured to confirm the qualitative results. DCX-positive neurons formed a narrower band in NBW than in LBW (38.3 ± 2.1 µm versus 46.2 ± 3.5 µm, *p* = 0.024), suggesting that the NBW animals had fewer immature neurons because they were already migrating to the other areas. When differentiating the animals according to whether their mothers had received HTX or not, it was observed that the thickness of LBW animals whose mothers received HTX had a tendency to be lower than that of animals from the CTRL group (38.9 ± 2.6 µm versus 53.5 ± 1.2 µm, *p* = 0.063, panels d2 and b2). The same occurred in NBW animals; those that received HTX presented a narrower band compared with the control group (29.8 ± 1.3 µm versus 43.0 ± 2.6 µm, *p* = 0.001, panels c2 and a2).

**Figure 3:**
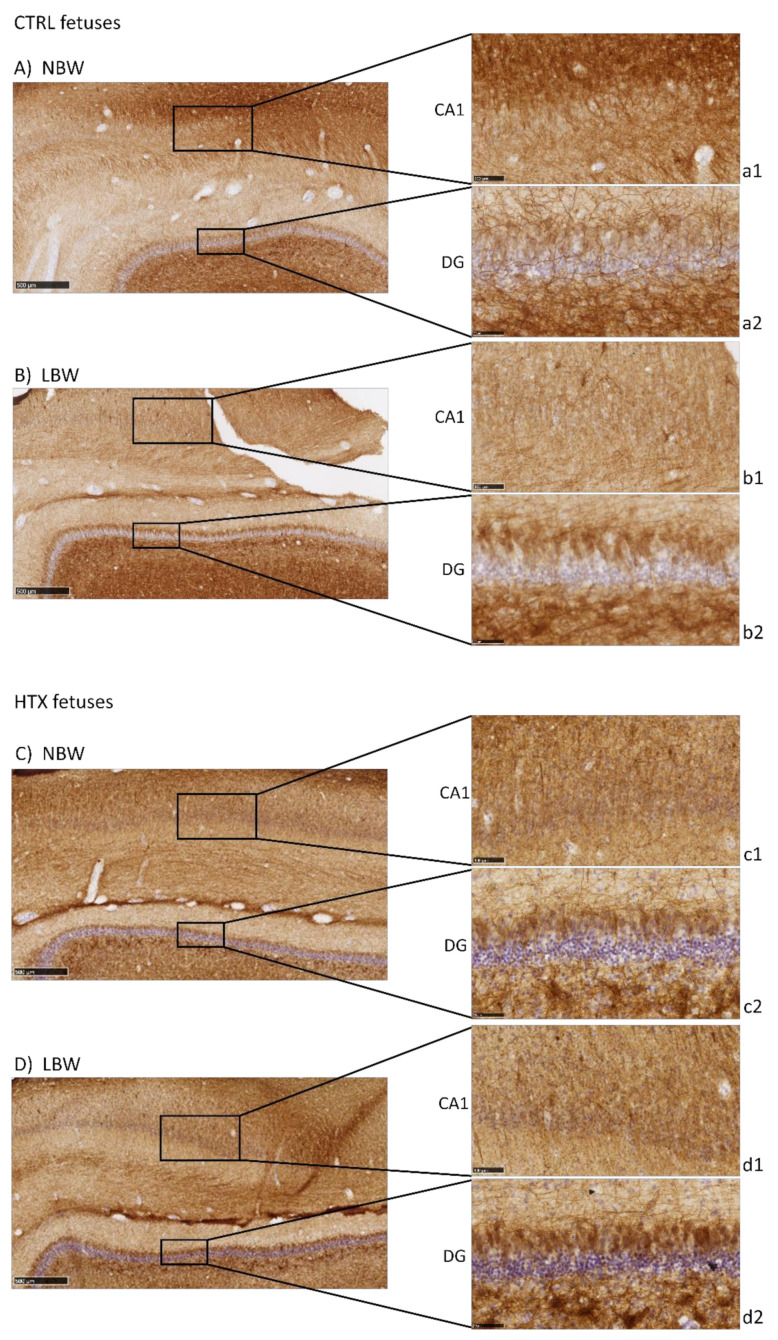
Effect of IUGR on neurofilaments (NFT) immunostaining on the hippocampus of fetuses whose mothers were not HTX-supplemented (**A**,**B**) or were HTX-supplemented (**C**,**D**). Panels are magnifications of the CA1 (**a1**–**d1**) and DG (**a2**–**d2**) areas shown using black boxes. Scale bars: 500 µm (**A**–**D**), 100 µm (**a1**–**d1**), and 50 µm (**a2**–**d2**).

Neurofilament protein immunostaining (NFT) provided specific immunostaining of axons in mature neurons. Positive staining is observed as a mesh of filaments, preventing the quantification of the experiment. Results were similar to those of NeuN in CA1 and DG, with a more intense labeling in the NBW fetuses compared with LBW (Figure 3), only in the control group. In LBW animals whose mothers had received HTX supplementation, a more intense staining was observed, thus suggesting again that HTX was able to reverse the effects produced by IUGR. Similarly, more connectivity through the granular cell layer was observed in NBW than in LBW individuals in the control groups, whereas there were no differences between the HTX groups.

### 3.2. Effects of Maternal Supplementation with HTX on the Neurotransmitter Profile in Several Brain Areas of NBW and LBW Fetuses 

The effect of weight and maternal supplementation with HTX on the NT profile of the hippocampus was analyzed and it is shown in Table 2. No differences were seen between NBW and LBW animals, but an effect of HTX on several NTs, especially from the dopaminergic pathway, was observed as described in our previous work [22]. Nevertheless, an interaction between HTX treatment and body weight was observed in 5-HT, a tendency in 5-HIAA, and consequently an effect in total indoleamines (INDtotal), indicating that maternal supplementation with HTX differentially affected NBW and LBW offspring. When analyzing in detail this interaction, higher values of 5-HT and, therefore, of INDtotal were found in LBW fetuses than in NBW fetuses of the control group (*p* = 0.015 and *p* = 0.016, respectively, in the pairwise comparison). Differences due to body weight disappeared in the HTX-treated group (*p* = 0.285 and *p* = 0.305, respectively), indicating that the effect caused by IUGR on indoleamines was not observed in the case of maternal supplementation with HTX.

Finally, interactions between the body weight and sex of fetuses were analyzed but not found to be significant for any variable (Table 3).

Neurotransmitters were also quantified in amygdalae and the prefrontal cortex. Differences between control and HTX groups were observed as described before [22], but no effect of body weight was seen on any of the NTs or brain area (Supplementary Table S2). Similarly, no effect of sex or interaction between sex and body weight was observed in these two brain areas, except for sex and NA in the amygdalae (Supplementary Table S3).

## 4. Discussion

### 4.1. Effects on Hippocampal Development

The hippocampus is related to memory processes, cognitive functions, learning capacities, and motor skills, which are essential for normal neurological development [27]. Several studies indicate that the hippocampus is very vulnerable to hypoxia, malnutrition, and altered micronutrient supply, which are present in IUGR [28]. In the hippocampus, pyramidal neurons from CA originate in the second half of the embryonic life. The multiplying neuroblasts migrate from the ventricular zone to their final target region and the route of migration is short because the hippocampus closely follows the curve of the ventricle. Future CA1 neurons form cell rows perpendicular to the ventricular germinal layer. At birth, the pyramidal layer is thick but it becomes thinner postnatally. On the other side, the generation of the granule cells in the DG starts at the middle of gestation but continues long into the postnatal period and, at a reduced level, into adulthood [29].

In the present work, labeling with NeuN and NFT antibodies, both markers of mature neurons, indicated that there is a neuronal deficit in the CA1 and DG areas of the hippocampi of LBW fetuses (affected by IUGR). Inversely, DCX labeling suggested that LBW fetuses had a higher number of immature and disorganized neurons than NBW fetuses. Neuron precursor cells express the DCX protein and, once they receive a synaptic signal, they extend dendrites and axons and express a series of markers from mature neurons (e.g., NeuN) [30]. Thus, our results indicate that cell differentiation proceeds more slowly in LBW than in NBW animals. The changes observed in LBW fetuses are especially relevant in DG, an area where new neurons are generated continuously throughout the individual’s life [31,32].

These results confirm those previously described in the literature. Thus, MRI studies in humans confirm that the hippocampus is susceptible to IUGR, showing a reduced volume in neonatal periods [28]. In laboratory animals, it has been shown that IUGR affects the total number of pyramidal neurons in CA1 [33] and that the surviving neurons present an abnormal axonal and dendritic morphology as well as impaired connectivity between them [34,35]. Furthermore, it has been described that IUGR can affect the volume and number of neurons [5,36] as well as neurotransmission [37], thus altering the development of the hippocampus. Taken together, our results indicate that the hippocampal maturation process is slower in LBW fetuses, which present a greater number of immature neurons. Due to the relevance of the correct development of the hippocampus and its important role in various functions [38], our results support other research findings indicating that the morphological changes observed in individuals affected by IUGR may be associated with defects in cognitive function, such as memory and learning, in humans or animal models [1,5,13,39]. In a porcine model similar to ours, it was found that the IUGR newborn piglet brain displays less NeuN-positive cells in the parietal cortex, together with other alterations affecting glial morphology, neuronal damage, white matter disruption, and inflammation [40].

Our results indicate that HTX may counteract the deleterious effect of growth restriction. Importantly, maternal treatment with HTX caused an increase in the number of NeuN-positive neurons and a decrease in the number of DCX-positive neurons in LBW fetuses, thus appearing more similar to NBW individuals. In NBW fetuses, HTX caused milder effects, which were observed only as a significant decrease in cell nuclei size in NeuN-stained samples. That is, the effects of maternal supplementation with HTX affect NBW and LBW fetuses in the same way, but the effects are more significant in LBW fetuses. Therefore, treatment of the mothers with HTX reversed the effects of IUGR in the hippocampus of the LBW fetuses. As a consequence, the differences observed between NBW and LBW individuals in the control groups disappear or are weaker when comparing HTX-NBW and HTX-LBW groups. This would be in agreement with reports indicating that maternal HTX administration improves neurogenesis and cognitive function in the offspring of prenatally stressed rats [41]. It is widely accepted that the protective role of a Mediterranean diet during pregnancy for the health of the mother and child is mainly due to the antioxidants supplied by components of this diet, such as olive oil [42,43], and it is important to note that HTX is able to cross the blood–brain barrier (BBB), thus reaching the brain at physiologically relevant concentrations [44].

### 4.2. Effects on the Neurotransmitter Profile in the Hippocampus

The effect of IUGR in relation to HTX supplementation of the mother’s diet primarily altered the serotonergic pathway in the hippocampus. There is a growing body of evidence indicating that indoleamines, especially serotonin, are crucial for the modulation of neurodevelopmental processes, and therefore in the development of the fetal brain [45]. Thus, it has been described that changes in the serotoninergic system during the prenatal stages can contribute to an alteration in development through fetal programming mechanisms and an increased risk of psychiatric diseases during childhood and adulthood [46]. During postnatal life, indoleamines are involved in such important aspects such as food intake, mood, and social interactions [47]. Similar to our results, other authors have also described that IUGR significantly increases the levels of 5-HT and its metabolite 5-HIAA, affecting the serotonergic pathway in rodent animal models [48,49].

It is possible that the small differences observed in neurotransmitter levels between LBW and NBW fetuses are related to asymmetric growth and prioritization of brain development during IUGR. However, alterations in the concentration and metabolism of indoleamines in the limbic system (mainly in the hippocampus) can compromise adequate neural function. Specifically, in humans, individuals with LBW have been found to have a higher incidence of behavioural disorders related to mobility, cognition, memory, and neuropsychological functions [50,51].

Again, an interaction between body weight and maternal supplementation with HTX was observed since the effect caused by IUGR on indoleamines was not observed in the treated group. In the pairwise comparison, LBW individuals showed different indoleamine concentrations to NBW individuals in the control groups, but these differences disappear between HTX-supplemented groups.

On the other hand, treatment of the mothers with HTX mainly affected the dopaminergic pathway in the hippocampus and that could be related to the fact that DA metabolism is a major source of intracellular ROS production [52]. HTX has a potent ROS scavenger capacity and it can induce several antioxidant enzymatic systems in the cell and maintain high levels of reduced glutathione [11,41,53]. Other possibilities would be enzymatic mechanisms by acting on Phase II enzymes, COMT, or other dopamine-related enzymes as discussed in our previous work [22].

## 5. Conclusions

In 100-day-old fetuses, IUGR conditions altered the immunohistochemical characteristics of the hippocampus as well as the neurotransmitter profile. Supplementation with HTX was able to reverse these changes, leading both characteristics to values more similar to those in NBW pigs. The results of the present study support the usefulness of maternal supplementation with HTX to improve prenatal development in a swine model of IUGR pregnancies.

## Figures and Tables

**Table 1:** Effect of body weight and supplementation of the maternal diet with hydroxytyrosol (HTX) on NeuN immunostaining in the CA1 and DG areas of the hippocampus of 100-day-old fetuses.

			NBW	LBW	*p*-Values		
					Treatment	Body Weight	Interaction
**CA1**	**Cell Count**	CTRL	143.06 ± 9.02 ^Aa^	87.25 ± 11.40 ^Ab^	<0.001	0.004	0.035
		HTX	170.86 ± 9.69 ^Ba^	162.00 ± 6.04 ^Ba^			
	**Mean size (µm^2^)**	CTRL	1352.44 ± 247.19 ^Aa^	2961.48 ± 676.06 ^Ab^	<0.001	0.013	0.015
		HTX	817.85 ± 61.87 ^Aa^	834.88 ± 44.94 ^Ba^			
	**Area (%)**	CTRL	33.48 ± 1.29 ^Aa^	41.63 ± 2.76 ^Ab^	<0.001	0.007	0.033
		HTX	30.07 ± 0.99 ^Aa^	31.07 ± 1.14 ^Ba^			
**DG**	**Cell Count**	CTRL	79.91 ± 2.25 ^Aa^	79.33 ± 4.55 ^Aa^	0.045	0.831	0.719
		HTX	86.50 ± 3.64 ^Aa^	88.75 ± 6.03 ^Aa^			
	**Mean size (µm^2^)**	CTRL	1449.37 ± 51.72 ^Aa^	1620.75 ± 116.92 ^Aa^	<0.001	0.677	0.014
		HTX	1248.78 ± 62.85 ^Ba^	1009.78 ± 48.95 ^Ba^			
	**Area (%)**	CTRL	26.34 ± 0.55 ^Aa^	28.76 ± 0.61 ^Ab^	<0.001	0.282	<0.001
		HTX	24.49 ± 0.49 ^Ba^	20.52 ± 0.76 ^Bb^			

The results are presented as the mean ± SE. The superscripts and *p*-values in bold show the significant differences. Different capital letters (Aa = Ab ≠ Ba = Bb) represent significant differences between treatments for the same group of body weight (*p* < 0.05). Different lowercase letters (Aa = Ba ≠ Ab = Bb) represent significant differences between groups of body weight for the same treatment (*p* < 0.05). Statistical significance was determined by UNIANOVA with Tukey’s adjustment.

**Table 2:** Concentration of neurotransmitters and their metabolites in the hippocampus of 100-day-old fetuses as a function of maternal supplementation with HTX according to their body weight.

		NBW	LBW	*p*-Values		
				Treatment	Body Weight	Interaction
**NA**	CTRL	57.82 ± 3.13 ^Aa^	73.47 ± 10.71 ^Aa^	<0.001	0.383	0.235
	HTX	81.20 ± 4.07 ^Ba^	81.53 ± 13.24 ^Aa^			
**DA**	CTRL	95.86 ± 10.99 ^Aa^	106.74 ± 13.06 ^Aa^	0.788	0.414	0.374
	HTX	88.85 ± 5.18 ^Aa^	85.58 ± 9.92 ^Aa^			
**DOPAC**	CTRL	53.72 ± 7.22 ^Aa^	57.24 ± 16.99 ^Aa^	<0.001	0.703	0.824
	HTX	76.71 ± 8.73 ^Ba^	97.42 ± 32.47 ^Aa^			
**HVA**	CTRL	197.59 ± 14.50 ^Aa^	259.56 ± 31.19 ^Ab^	<0.001	0.076	0.404
	HTX	270.10 ± 14.04 ^Ba^	306.08 ± 46.88 ^Aa^			
**DOPtotal**	CTRL	347.17 ± 24.87 ^Aa^	423.54 ± 49.48 ^Aa^	0.001	0.140	0.480
	HTX	435.66 ± 19.97 ^Ba^	489.08 ± 78.44 ^Aa^			
**CATtotal**	CTRL	404.99 ± 26.96 ^Aa^	497.00 ± 59.46 ^Aa^	<0.001	0.151	0.433
	HTX	516.86 ± 22.09 ^Ba^	570.61 ± 88.46 ^Aa^			
**5-HT**	CTRL	167.74 ± 8.29 ^Aa^	221.61 ± 17.48 ^Ab^	0.017	0.482	0.019
	HTX	216.95 ± 11.75 ^Ba^	195.07 ± 31.84 ^Aa^			
**5-HIAA**	CTRL	85.51 ± 4.57 ^Aa^	102.58 ± 8.98 ^Aa^	0.155	0.461	0.096
	HTX	94.76 ± 3.07 ^Ba^	90.99 ± 12.18 ^Aa^			
**INDtotal**	CTRL	253.25 ± 11.84 ^Aa^	324.19 ± 23.37 ^Ab^	0.026	0.468	0.021
	HTX	311.71 ± 13.64 ^Ba^	286.05 ± 43.64 ^Aa^			

Concentrations are presented as the mean ± SE. Units are ng/g tissue. The rows divide fetuses as a function of their body weight (NBW or LBW). The columns show the neurotransmitters and metabolites as a function of the treatment (CTRL or HTX). The superscripts and *p*-values in bold indicate significant differences. Different capital letters (Aa = Ab ≠ Ba) represent significant differences between treatments for the same group of body weight (*p* < 0.05). Different lowercase letters (Aa = Ba ≠ Ab) represent significant differences between groups of body weight for the same treatment (*p* < 0.05). NA, noradrenalin; DA, dopamine; DOPAC, 3,4-dihydroxyphenyl acetic acid; HVA, homovanillic acid; 5-HT, serotonin/5-hydroxytryptamine; 5-HIAA, 5-hydroxyindoleacetic acid; DOP total, total dopaminergic neurotransmitters; CAT total, total catecholaminergic neurotransmitters; IND total, total serotoninergic neurotransmitters. Statistical significance was determined by UNIANOVA with Tukey’s adjustment.

**Table 3:** Concentration of neurotransmitters and their metabolites in the hippocampus of 100-day-old fetuses as a function of body weight and sex.

		Females	Males	*p*-Values		
				Body Weight	Sex	Interaction
**NA**	NBW	68.58 ± 3.56	68.76 ± 4.65	0.421	0.915	0.990
	LBW	75.95 ± 10.79	77.03 ± 12.90			
**DA**	NBW	93.89 ± 9.38	90.81 ± 7.82	0.316	0.546	0.656
	LBW	91.75 ± 12.57	105.85 ± 13.58			
**DOPAC**	NBW	65.19 ± 7.76	63.23 ± 8.49	0.928	0.413	0.534
	LBW	81.90 ± 25.69	62.72 ± 21.23			
**HVA**	NBW	228.31 ± 13.48	235.27 ± 18.19	0.107	0.715	0.619
	LBW	293.77 ± 39.25	260.24 ± 35.56			
**DOPtotal**	NBW	387.38 ± 21.60	389.30 ± 27.33	0.170	0.735	0.700
	LBW	467.42 ± 60.54	428.80 ± 61.21			
**CATtotal**	NBW	455.96 ± 23.58	458.07 ± 30.75	0.185	0.747	0.753
	LBW	543.38 ± 70.25	505.83 ± 72.12			
**5-HT**	NBW	188.55 ± 10.20	193.35 ± 11.11	0.316	0.819	0.576
	LBW	214.33 ± 21.08	208.98 ± 25.20			
**5-HIAA**	NBW	88.64 ± 3.18	91.43 ± 5.30	0.314	0.427	0.371
	LBW	103.44 ± 8.53	93.03 ± 11.72			
**INDtotal**	NBW	277.19 ± 12.29	284.79 ± 15.06	0.300	0.693	0.514
	LBW	317.76 ± 28.64	302.01 ± 34.02			

Concentrations are presented as the mean ± SE. Units ng/g tissue. The rows divide fetuses as a function of their sex (females or males). The columns show the neurotransmitters and metabolites as a function of their body weight (NBW or LBW). NA, noradrenalin; DA, dopamine; DOPAC, 3,4-dihydroxyphenyl acetic acid; HVA, homovanillic acid; 5-HT, serotonin/5-hydroxytryptamine; 5-HIAA, 5-hydroxyindoleacetic acid; DOP total, total dopaminergic neurotransmitters; CAT total, total catecholaminergic neurotransmitters; IND total, total serotoninergic neurotransmitters. Statistical significance was determined by UNIANOVA with Tukey’s adjustment.

## Data Availability

Data is contained within the article.

## Supplementary Materials

The following are available online at www.mdpi.com/article/10.3390/antiox10101505/s1, Table S1: Mean body weight and number of individuals included in the immunohistochemical analysis. Table S2: Concentration of neurotransmitters and their metabolites in the amygdala and prefrontal cortex as a function of maternal supplementation with or without HTX in 100-day-old fetuses according to their body weight. Table S3: Concentration of neurotransmitters and their metabolites in the amygdala and prefrontal cortex as a function of body weight and sex in fetuses at 100 days’ gestation.

## References

[1] Sharma D., Shastri S., Sharma P. (2016). Intrauterine Growth Restriction: Antenatal and Postnatal Aspects. Clin. Med. Insights. Pediatr..

[2] Wollmann H. (1998). Intrauterine growth restriction: Definition and etiology. Horm. Res..

[3] Cohen E., Baerts W., van Bel F. (2015). Brain-Sparing in Intrauterine Growth Restriction: Considerations for the Neonatologist. Neonatology.

[4] Garcia-Contreras C., Vazquez-Gomez M., Astiz S., Torres-Rovira L., Sanchez-Sanchez R., Gomez-Fidalgo E., Gonzalez J., Isabel B., Rey A., Ovilo C. (2017). Ontogeny of Sex-Related Differences in Foetal Developmental Features, Lipid Availability and Fatty Acid Composition. Int. J. Mol. Sci..

[5] Miller S., Huppi P., Mallard C. (2016). The consequences of fetal growth restriction on brain structure and neurodevelopmental outcome. J. Physiol..

[6] Rashid C.S., Bansal A., Simmons R.A. (2018). Oxidative stress, intrauterine growth restriction, and developmental programming of type 2 diabetes. Physiology.

[7] Aljunaidy M.M., Morton J.S., Cooke C.L.M., Davidge S.T. (2017). Prenatal hypoxia and placental oxidative stress: Linkages to developmental origins of cardiovascular disease. Am. J. Physiol.-Regul. Integr. Comp. Physiol..

[8] Richter H.G., Camm E.J., Modi B.N., Naeem F., Cross C.M., Cindrova-Davies T., Spasic-Boskovic O., Dunster C., Mudway I.S., Kelly F.J. (2012). Ascorbate prevents placental oxidative stress and enhances birth weight in hypoxic pregnancy in rats. J. Physiol..

[9] Wang Y., Fu W., Liu J. (2016). Neurodevelopment in children with intrauterine growth restriction: Adverse effects and interventions. J. Matern. Neonatal Med..

[10] Yzydorczyk C., Armengaud J.B., Peyter A.C., Chehade H., Cachat F., Juvet C., Siddeek B., Simoncini S., Sabatier F., Dignat-George F. (2017). Endothelial dysfunction in individuals born after fetal growth restriction: Cardiovascular and renal consequences and preventive approaches. J. Dev. Orig. Health Dis..

[11] Robles-Almazan M., Pulido-Moran M., Moreno-Fernandez J., Ramirez-Tortosa C., Rodriguez-Garcia C., Quiles J.L., Ramirez-Tortosa M.C. (2018). Hydroxytyrosol: Bioavailability, toxicity, and clinical applications. Food Res. Int..

[12] Bertelli M., Kiani A.K., Paolacci S., Manara E., Kurti D., Dhuli K., Bushati V., Miertus J., Pangallo D., Baglivo M. (2020). Hydroxytyrosol: A natural compound with promising pharmacological activities. J. Biotechnol..

[13] Zheng A., Li H., Cao K., Xu J., Zou X., Li Y., Chen C., Liu J., Feng Z. (2015). Maternal hydroxytyrosol administration improves neurogenesis and cognitive function in prenatally stressed offspring. J. Nutr. Biochem..

[14] de Pablos R.M., Espinosa-Oliva A.M., Hornedo-Ortega R., Cano M., Arguelles S. (2019). Hydroxytyrosol protects from aging process via AMPK and autophagy; a review of its effects on cancer, metabolic syndrome, osteoporosis, immune-mediated and neurodegenerative diseases. Pharmacol. Res..

[15] Óvilo C., González-Bulnes A., Benítez R., Ayuso M., Barbero A., Pérez-Solana M.L., Barragán C., Astiz S., Fernández A., López-Bote C. (2014). Prenatal programming in an obese swine model: Sex-related effects of maternal energy restriction on morphology, metabolism and hypothalamic gene expression. Br. J. Nutr..

[16] Gonzalez-Bulnes A., Astiz S., Parraguez V.H., Garcia-Contreras C., Vazquez-Gomez M. (2016). Empowering Translational Research in Fetal Growth Restriction: Sheep and Swine Animal Models. Curr. Pharm. Biotechnol..

[17] Vazquez-Gomez M., Garcia-Contreras C., Torres-Rovira L., Pesantez J.L., Gonzalez-Añover P., Gomez-Fidalgo E., Sanchez-Sanchez R., Ovilo C., Isabel B., Astiz S. (2017). Polyphenols and IUGR pregnancies: Maternal hydroxytyrosol supplementation improves prenatal and early-postnatal growth and metabolism of the offspring. PLoS ONE.

[18] Vazquez-Gomez M., Heras-Molina A., Garcia-Contreras C., Pesantez-Pacheco J.L., Torres-Rovira L., Martinez-Fernandez B., Gonzalez J., Encinas T., Astiz S., Ovilo C. (2019). Polyphenols and IUGR pregnancies: Effects of maternal hydroxytyrosol supplementation on postnatal growth, metabolism and body composition of the offspring. Antioxidants.

[19] Garcia-Contreras C., Vazquez-Gomez M., Barbero A., Pesantez J.L., Zinellu A., Berlinguer F., Gonzalez-Añover P., Gonzalez J., Encinas T., Torres-Rovira L. (2019). Polyphenols and iugr pregnancies: Effects of maternal hydroxytyrosol supplementation on placental gene expression and fetal antioxidant status, dna-methylation and phenotype. Int. J. Mol. Sci..

[20] Garcia-Contreras C., Vazquez-Gomez M., Pardo Z., Heras-Molina A., Pesantez J.L., Encinas T., Torres-Rovira L., Astiz S., Nieto R., Ovilo C. (2019). Polyphenols and IUGR pregnancies: Effects of maternal hydroxytyrosol supplementation on hepatic fat accretion and energy and fatty acids profile of fetal tissues. Nutrients.

[21] Heras-Molina A., Pesantez-Pacheco J.L., Astiz S., Garcia-Contreras C., Vazquez-Gomez M., Encinas T., Óvilo C., Isabel B., Gonzalez-Bulnes A. (2020). Maternal supplementation with polyphenols and omega-3 fatty acids during pregnancy: Effects on growth, metabolism, and body composition of the offspring. Animals.

[22] Yeste N., Valent D., Arroyo L., Vázquez-Gómez M., García-Contreras C., Pumarola M., González-Bulnes A., Bassols A. (2021). Polyphenols and IUGR Pregnancies: Effects of the Antioxidant Hydroxytyrosol on Brain Neurochemistry and Development in a Porcine Model. Antioxidants.

[23] Whittemore C.T., Hazzledine M.J., Close W.H. (2003). Nutrient Requirement Standards for Pigs.

[24] Gonzalez-Bulnes A., Ovilo C., Lopez-Bote C.J., Astiz S., Ayuso M., Perez-Solana M., Sanchez-Sanchez R., Torres-Rovira L. (2012). Gender-specific early postnatal catch-up growth after intrauterine growth retardation by food restriction in swine with obesity/leptin resistance. Reproduction.

[25] Gonzalez-Bulnes A., Astiz S., Ovilo C., Lopez-Bote C.J., Torres-Rovira L., Barbero A., Ayuso M., Garcia-Contreras C., Vazquez-Gomez M. (2016). Developmental Origins of Health and Disease in swine: Implications for animal production and biomedical research. Theriogenology.

[26] Arroyo L., Carreras R., Valent D., Peña R., Mainau E., Velarde A., Sabrià J., Bassols A. (2016). Effect of handling on neurotransmitter profile in pig brain according to fear related behaviour. Physiol. Behav..

[27] Kesner R.P., Lee I., Gilbert P. (2004). A behavioral assessment of hippocampal function based on a subregional analysis. Rev. Neurosci..

[28] Lodygensky G.A., Seghier M.L., Warfield S.K., Tolsa C.B., Sizonenko S., Lazeyras F., Hüppi P.S. (2008). Intrauterine Growth Restriction Affects the Preterm Infant’s Hippocampus. Pediatr. Res..

[29] Frotscher M., Seress L., Andersen P., Morris R., Amaral D., Bliss T., O’Keefe J. (2007). Morphological development of the hippocampus. The Hippocampus Book.

[30] von Bohlen Und Halbach O. (2007). Immunohistological markers for staging neurogenesis in adult hippocampus. Cell Tissue Res..

[31] Gonçalves J.T., Schafer S.T., Gage F.H. (2016). Adult Neurogenesis in the Hippocampus: From Stem Cells to Behavior. Cell.

[32] Eriksson P., Perfilieva E., Björk-Eriksson T., Alborn A., Nordborg C., Peterson D., Gage F. (1998). Neurogenesis in the adult human hippocampus. Nat. Med..

[33] Mallard C., Loeliger M., Copolov D., Rees S. (2000). Reduced number of neurons in the hippocampus and the cerebellum in the postnatal guinea-pig following intrauterine growth-restriction. Neuroscience.

[34] Dieni S., Rees S. (2003). Dendritic morphology is altered in hippocampal neurons following prenatal compromise. J. Neurobiol..

[35] Basilious A., Yager J., Fehlings M.G. (2015). Neurological outcomes of animal models of uterine artery ligation and relevance to human intrauterine growth restriction: A systematic review. Dev. Med. Child Neurol..

[36] Cumberland A.L., Palliser H.K., Rani P., Walker D.W., Hirst J.J. (2017). Effects of combined IUGR and prenatal stress on the development of the hippocampus in a fetal Guinea pig model. J. Dev. Orig. Health Dis..

[37] Morgane P.J., Mokler D.J., Galler J.R. (2002). Effects of prenatal protein malnutrition on the hippocampal formation. Neurosci. Biobehav. Rev..

[38] Dupret D., Revest J.-M., Koehl M., Ichas F., De Giorgi F., Costet P., Abrous D.N., Piazza P.V. (2008). Spatial relational memory requires hippocampal adult neurogenesis. PLoS ONE.

[39] Wixey J.A., Chand K.K., Colditz P.B., Bjorkman S.T. (2017). Review: Neuroinflammation in intrauterine growth restriction. Placenta.

[40] Wixey J.A., Lee K.M., Miller S.M., Goasdoue K., Colditz P.B., Tracey Bjorkman S., Chand K.K. (2019). Neuropathology in intrauterine growth restricted newborn piglets is associated with glial activation and proinflammatory status in the brain 11 Medical and Health Sciences 1109 Neurosciences. J. Neuroinflamm..

[41] Zheng A., Li H., Xu J., Cao K., Li H., Pu W., Yang Z., Peng Y., Long J., Liu J. (2015). Hydroxytyrosol improves mitochondrial function and reduces oxidative stress in the brain of *db/db* mice: Role of AMP-activated protein kinase activation. Br. J. Nutr..

[42] Al-Gubory K.H. (2013). Maternal nutrition, oxidative stress and prenatal devlopmental outcomes. Studies on Women’s Health.

[43] Mariscal-Arcas M., Monteagudo C., Olea-Serrano F. (2013). Diet quality in pregnancy: A focus on requirements and the protective effects of the Mediterranean diet. Diet Quality: An Evidence-Based Approach.

[44] Domínguez-Perles R., Auñón D., Ferreres F., Gil-Izquierdo A. (2017). Physiological linkage of gender, bioavailable hydroxytyrosol derivatives, and their metabolites with systemic catecholamine metabolism. Food Funct..

[45] Velasquez J.C., Goeden N., Bonnin A. (2013). Placental serotonin: Implications for the developmental effects of SSRIs and maternal depression. Front. Cell. Neurosci..

[46] St-Pierre J., Laurent L., King S., Vaillancourt C. (2016). Effects of prenatal maternal stress on serotonin and fetal development. Placenta.

[47] Berumen L.C., Rodríguez A., Miledi R., García-Alcocer G. (2012). Serotonin Receptors in Hippocampus. Sci. World J..

[48] Jensen A., Klonne H.J., Detmer A., Carter A.M. (1996). Catecholamine and serotonin concentrations in fetal guinea-pig brain: Relation to regional cerebral blood flow and oxygen delivery in the growth-restricted fetus. Reprod. Fertil. Dev..

[49] Manjarrez G.G., Magdaleno V.M., Chagoya G., Hernández J. (1996). Nutritional recovery does not reverse the activation of brain serotonin synthesis in the ontogenetically malnourished rat. Int. J. Dev. Neurosci..

[50] Bauer R., Walter B., Brust P., Füchtner F., Zwiener U. (2003). Impact of asymmetric intrauterine growth restriction on organ function in newborn piglets. Eur. J. Obstet. Gynecol. Reprod. Biol..

[51] Geva R., Leitner Y., Harel S. (2012). Children Born with Intrauterine Growth Restriction: Neurodevelopmental Outcome. Handbook of Growth and Growth Monitoring in Health and Disease.

[52] Meiser J., Weindl D., Hiller K. (2013). Complexity of dopamine metabolism. Cell Commun. Signal..

[53] Martin M.A., Ramos S., Granado-Serrano A.B., Rodriguez-Ramiro I., Trujillo M., Bravo L., Goya L. (2010). Hydroxytyrosol induces antioxidant/detoxificant enzymes and Nrf2 translocation via extracellular regulated kinases and phosphatidylinositol-3-kinase/ protein kinase B pathways in HepG2 cells. Mol. Nutr. Food Res..

